# Comparative Study of Different Drills for Bone Drilling: A Systematic Approach

**DOI:** 10.5704/MOJ.2007.016

**Published:** 2020-07

**Authors:** O Pazarci, Y Torun, A Ozturk, Z Oztemur

**Affiliations:** 1 Department of Orthopaedic and Traumatology, Cumhuriyet University, Sivas, Turkey; 2 Department of Electrical and Electronics Engineering, Sivas Cumhuriyet University, Sivas, Turkey

**Keywords:** bone drilling, orthopaedical drill characteristics

## Abstract

**Introduction:**

The performance of the drilling process depends on the characteristics of the drilling equipment and surgeon’s skill. To our knowledge, no research has focused on multi-parameter analysis of the dynamic behaviour of drills during the drilling process. This study aimed to characterise the physical changes and effects of different drills attached to a robotic arm during drilling of artificial bones in a standardised experimental setup.

**Material and Methods:**

Drilling processes using three brands of drills attached to a robotic arm were compared in terms of thrust force, vibration, noise level, speed deviation, and temperature. A standardised experimental setup was constructed, and measurement data were analysed statistically. Identical artificial bones were drilled 10 times with each drill.

**Results:**

Thrust force measurements, which varied through the cortex and medulla, showed expressive differences for each drill for maximum and mean values (p<0.001). Meaningful differences were obtained for mean vibration values and noise level (p<0.001). Speed variation measurements in drilling showed conspicuous differences with confident statistics (p<0.001). Induced temperature values were measured statistically for Drill 1, Drill 2, and Drill 3 as 78.38±11.49°C, 78.11±7.79°C, and 89.77±7.79°C, respectively.

**Conclusion:**

Thrust force and drill bit temperature were strongly correlated for each drill. Vibration values and noise level, which also had an influential relationship, were in the acceptable range for all experiments. Both thrust force and speed deviation information could be used to detect the drill bit status in the bone while drilling.

## Introduction

Bone fracture treatment involves bone drilling to fix fractured bone with specialised equipment. Bone is an organic, mineralised, and hard type of tissue. The drilling process is influenced by drill, shape, drill bit material, and bone structure^1^. In drilling operations, a thrust force is induced in the direction opposite to the drilling direction in relation to the cutting force of the drill bit. The cutting force is dependent on drill bit geometry, drill speed, and drill torque^2, 3^. Thrust force can be sensed by the surgical operator especially in transition sections in bones such as from a cortical to spongy bone and vice versa. Higher thrust force occurs on contact and in cortical bone, while lower values are obtained in spongy bone.

Motorised hand tools may cause hand vibration with values related to their power and mechanism. However, no study has reported that surgical drills can cause permanent or temporary damage to the surgical operator^4^. Vibration measurements consist of frequency, velocity, and velocity change of a mass. The term hand-arm vibration is defined as the vibration transmitted from a tool into the operator’s hands. Any device with vibration characteristics can cause damage to fingers, hands, and arms. The hand-arm vibration syndrome (HAVS) is a medical condition caused by working with vibrating tools or machinery. Vibration injuries are divided into three subgroups: neurological, vascular, and musculoskeletal disorders^5^.

Sound is a pressure variation wave that can be detected by the human ear. When dealing with electromechanical equipment in surgery, the surgical drill is the main source of noise which causes pressure changes in the air. Noise can be defined as an excessive or unwanted sound that potentially causes loss of attention. Thus, more silent surgical drill operation may be preferred for comfortable surgical operations^6^. Sound pressure level is commonly measured by microphone which converts sound pressure values to electrical voltage values.

The friction between the bone and rotary drill bit induces temperature increases in both the drill bit and drilled bone. It is an undesirable situation that causes not only deformation of the drill bit but also bone necrosis, which negatively influences bone fracture healing. The temperature depends on bit geometry, friction coefficient of the bone tissue, feed rate of drilling, drill speed, and cutting force produced by the drill bit^7, 8^.

Another condition encountered in drilling in surgical operations is the battery capacity, which is a measurement of energy stored in the battery. The unit milliampere-hour is an indication of the discharge time of the battery at the rated voltage. State-of-art, larger battery capacity indicates a heavier battery weight. The total weight of the drill consists of the drive, motor and gear box, electronic controller, drill chunk, and battery.

Some valuable research focuses on discovering the relationship between drilling parameters and thermal necrosis^9^. The effect of drill bit geometry, diameter, and material was also analysed in some recent works^10^. However, to the best of our knowledge, no research has focused on multi-parameter analysis of the dynamic behaviour of drills during the drilling process. An orthopaedic surgeon must know the characteristics of the drill when drilling a bone because drilling is not a benign procedure^11^. Thus, this study aimed to characterise the physical changes and effects of different drills attached to a robotic arm during drilling of artificial bones in a standardised experimental setup.

## Materials and Methods

**Fig. 1:**
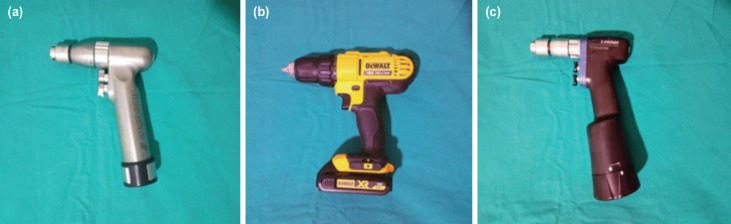
Drills used in experiments: (a) Drill 1: Aesculap^®^ Acculan 3. (b) Drill 2: Dewalt^®^ (c) Drill-3: Synthes TRS Modular Drive^®^.

**Fig. 2:**
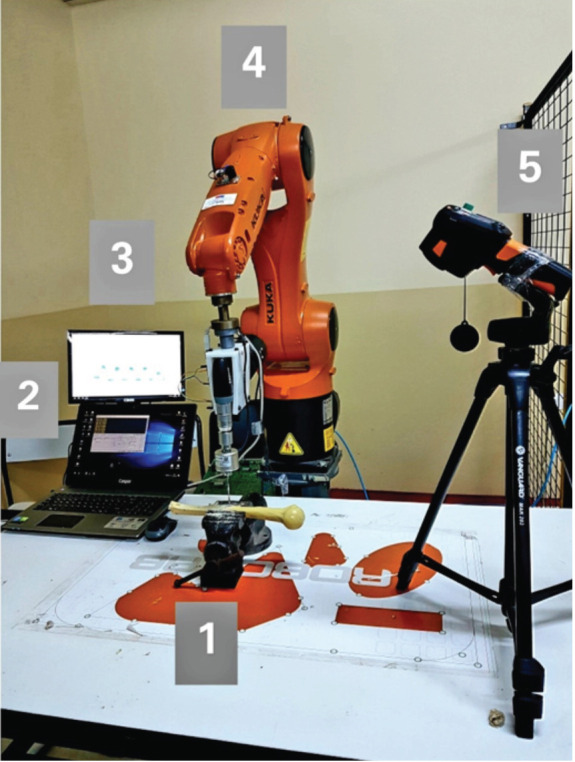
Experimental setup: (1) Compressed humerus sawbone [Synbone^®^, Swiss] in a clamp. (2) Computer where data and measurements are saved. (3) Drill attachment, measuring sensors and drill. (4) Robotic arm [Kuka Kr900^®^, Kuka Roboter GMBH]. (5) Thermal camera [Testo 869^®^].

The method and experimental setup are summarised in (Table I). Three different drills, namely, Aesculap^®^ Acculan 3Ti Tuttlingen/Germany (Drill 1), DeWalt^®^ DCD771C2 Pennsylvania/USA (Drill 2), and Synthes TRS Modular Drive^®^ Oberdorf/Switzerland (Drill 3), were used in our experiments (Fig. 1). Battery capacity, weight, and dimensions are listed in (Table II). To compare the drilling performance of each drill, experiments were performed on identical artificial bones, which were the humerus sawbone product of Synbone^®^ (Swiss) (Fig. 2).

**Table I:** Methodology Flowchart

**1. Drills, Artificial Bones and Drill Bit**
Drills: Aesculap® Acculan 3 (Drill-1), Dewalt^®^ (Drill-2) and Synthes TRS Modular Drive^®^ (Drill-3) Artificial Bones; humerus sawbone (Synbone^®^, Swiss) Drill Bit: 3.5 mm diameter twisted drill bit (Orcer^®^)
**2. Robotic Arm**
All drills were attached to the end effector of a six-axis Robotic Arm (Kuka Kr900^®^, Kuka Roboter GMBH) Robot was programmed to drill 10 holes with KRL (Kuka Robot Language).
**3. Measurement System**
Thrust force measurement: Loadcell transducer (TE Connectivity Measurement Specialties^®^, FC2231-0000-0100-L). Measurement of Vibration: 3-axis accelerometer and gyro sensor module (TDK InvenSense^®^, MPU6050). Noise level measurement: Electret condense microphone (OEM, XF- 18D). Rotational Speed: Optical Encoder (Opkon^®^, PRI 50H6 LTP 100 Z). Temperature of Drill Bit: Thermal Camera (Testo^®^, Testo 869). Data Acquisition System: 16-bit resolution-32 channels Data Acquisition System (Measurement Computing^®^, DAS 6025).

**Table II:** Battery voltage, battery capacity, weight and dimension features of drills.

	Units	Drill-1	Drill-2	Drill-3
		Aesculap Acculan 3	DeWalt DCD771C2	Synthes TRS Modular Drive
Battery Voltage	Voltage	9.6	18	25.2
Battery Capacity	Milliampere Hours	1950	1500	1282
Weight	Gram	3501	1585	3781
Dimension	Centimeter	20.69x23.03	21.9x19.1	21.99x30.32

To obtain standard test conditions, all drills were attached to the end effector of a six-axis robotic arm [Kuka Kr900^®^, Kuka Roboter GMBH] sequentially, with the same feed rates and axial forces. The robot (Fig. 2) is programmed to drill 10 holes with a distance of 10mm into each bone. The drill geometry was chosen as a twisted drill bit with 3.5mm diameter.

Thrust force measurement was performed by a load cell transducer unit that was attached between the drill and the robot flange to measure diagonal forces. Maximum thrust force is the arithmetical mean and standard deviation of maximum thrust forces measured for each hole. The average thrust force was specified as the mean and standard deviation of force measurements during the 10-hole drilling process.

For measuring robotic hand-held drill vibration, an inertial measurement system was used with a three-axis accelerometer for directional acceleration and a three-axis gyro for angular rate measurement. A micro electromechanical system inertial measurement sensor, MPU6050, was attached to the hand grip of the drill to measure vibration during drilling and free run cases. The sensor was able to measure acceleration up to ±16g and angular rate up to 2000°/second. Vibration measurement was performed as measurement of each axis and scalar magnitude quantity of the three-axis vibration. Vibration of the free run was measured when the drill was running freely instead of drilling bone. Vibration of drilling on bone measurement was performed during drilling bone.

For acoustic pressure measurement, an electret condenser microphone, OEM XF-18D, was used. In the experiment, a - 62 dBV sensitivity condense microphone was attached to the robotic gripper to measure the noise level of the operation during both free run and bone drilling cases. Noise level measurement was the arithmetic mean of noise levels for each 10-hole drilling process.

No drill had a built-in speed measurement device, so rotational speed was measured by a hollow shaft incremental optical encoder using PRI 50H6 LTP 100 Z series [Opkon Electronic].

The temperature of the drill bit cap was measured after drilling each hole using a thermal camera [Testo 869^®^]. The camera local focus was set to 50cm from the center of the saw bone sample. Ten measurements were performed to obtain drill bit cap temperature for each experiment.

To obtain speed variations during the drilling process, speed measurement was performed during the drilling process for both single hole speed variations and total 10-hole drilling speed variation.

Two brands of orthopaedic drills and one commercial hand drill with parameters defined in upper section were attached to drill 10 holes in three identical sawbone samples. The drilling process was performed by a robotic arm with the same feed rate for each brand drill. The humerus sawbone shaft double cortex was drilled. Sawbone samples were fixed with a laboratory clamp while the drilling process was performed (Fig. 2).

Signal processing and visualisation tasks were performed with 2.4 GHz, i7-4700HQ processor, 16 GB RAM computer with 16-bit resolution 32-channel data acquisition system board. Data were evaluated with SPSS version 22 software package [SPSS Inc., Chicago, IL, USA]. The Kruskal-Wallis H and Mann-Whitney tests were used to compare data groups. P values less than 0.05 were considered significant.

## Results:

**Fig. 3:**
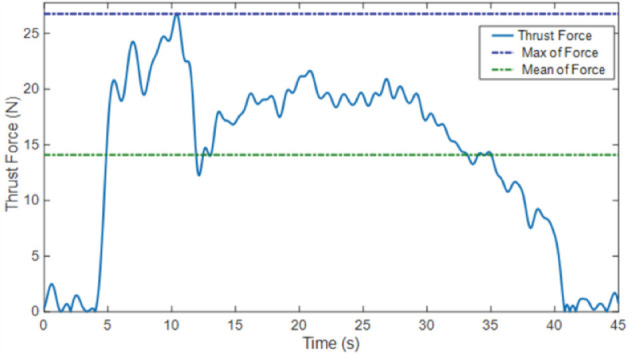
The force variation when the drill approaches the bone and passed through the proximal cortex – medulla – distal cortex (Drill 3).

Physical properties such as size, dimension weight, and battery capacity of the three drills are shown in (Table II). Thrust force measurements showed expressive differences for each drill (p<0.001). The highest thrust force values for both maximum thrust force and mean thrust force measurements were obtained for Drill 3. The thrust force variation during drilling of two cortices and medulla is shown in (Fig. 3) for one-hole drilling.

**Table III:** Thrust Force, Vibration, Noise Level and speed deviation characteristic

	Drill-1			Drill-2		Drill-3		p*	p**
	Aesculap Acculan 3			DeWalt DCD771C2		Synthes TRS Modular Drive			
Comparison Criteria	Units	Mean	Std	Mean	Std	Mean	Std		
Max Thrust Force	N	11.37	0.592	12.277	1.641	29.860	3.429	<0.001	<0.001^1^
Average Thrust Force	N	8.412	0.992	8.848	1.793	18.754	2.994	<0.001	<0.001^1^
Vibration Drilling on Bone	m/s^2^	1.225	0.041	1.9376	0.0655	1.1390	0.0052	<0.001	<0.001^1^
									<0.001^2^
Noise Level	Db	37.2614	0.4061	39.807	0.344	38.6799	0.058	<0.001	<0.001^1^
									<0.001^2^
Speed Deviation on drill operation	% RPM	0.1514	0.1713	0.6664	0.2359	0.1028	0.0379	<0.001	<0.001^3^

* Is the difference significant between groups?

** The drill causing the difference

^1^ Difference between Drill-3 and other drills

^2^ Difference between Drill-1 and Drill-2

^3^ Difference between Drill-2 and other drills

Meaningful differences were obtained for mean vibration values that were produced by drills during bone drilling. The lowest values were acquired for Drill 3, while the highest value was obtained with Drill 2. As shown in (Table III), the most silent drill was Drill 1, and the noisiest was Drill 2.

**Fig. 4:**
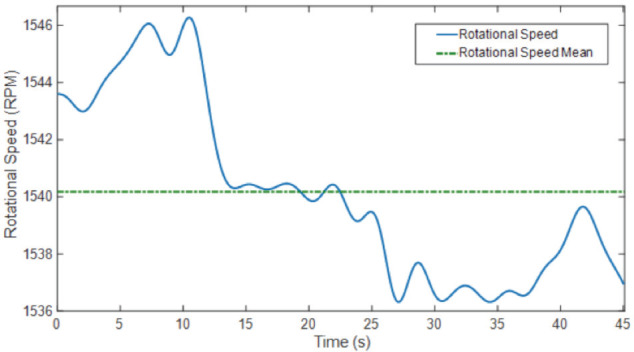
The speed variation when the drill approaches the bone and passes through the proximal cortex – medulla – distal cortex (Drill 2).

Speed deviation during drilling defines the speed variation of a drill when drilling different sections of the bone. The measured values for Drill 1 and Drill 3 were almost identical; however, there was a significant change in Drill 2, as shown in (Table III). Speed deviation for one-hole drilling is shown in (Fig. 4).

Vibration values at free runs without bone drilling are presented in (Table IV). According to the experiments, the lowest vibration values were measured for Drill 3, while the highest values were obtained with Drill 2.

**Table IV:** Vibration on free run, temperature of drill bit and Speed deviation for full operation measurements

	Drill-1			Drill-2		Drill-3	
	Aesculap Acculan 3			DeWalt DCD771C2		Synthes TRS Modular Drive	
Comparison Criteria	Units	Mean	Std	Mean	Std	Mean	Std
Vibration on free run	m/s2	1.239	0.1493	1.7689	0.4947	1.0816	0.3392
Temperature of Drill Bit	C0	78.38	11.49	78.11	7.38	89.77	7.79
Speed Deviation for full operation	% RPM	0,4719		6,3463		0,3018	

**Fig. 5:**
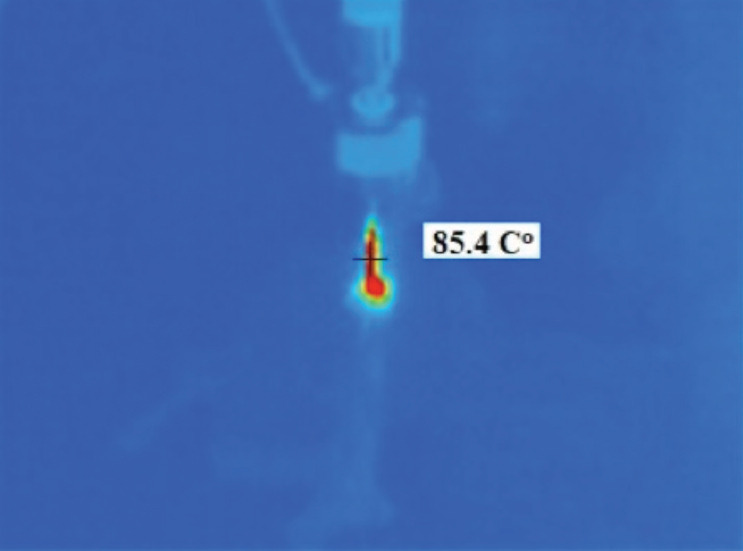
Thermal camera image of the drill bit reaching 85.4°C at the third hole (Drill 1).

**Fig. 6:**
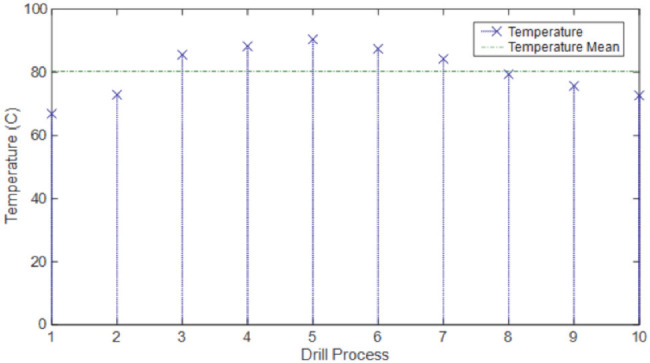
Temperature variation of the drill bit during drilling measured with a thermal camera (Drill 1). Each temperature measurement was made as the drill process was finished for each hole before beginning the next hole.

The recorded mean temperature values measured by a thermal camera in the entire drilling operations are shown in (Table IV). The highest temperature value of 89.77±7.79°C was measured with Drill 3, while the lowest temperature value of 78.11±7.38°C was measured with Drill 2. The thermal camera temperature image for Drill 1 is shown in (Fig. 5). Temperature variations from the first hole to the tenth hole after each drilling are shown in (Fig. 6).

Speed deviations for the entire 10-hole drilling process for each drill are given in (Table IV). Although speed variations for Drill 1 and Drill 3 were negligible, a significant speed deviation of 6.3463% RPM (Rounds per minute) was measured for Drill 2.

## Discussion

In our study, the characteristics of different drills using the same drill bit were compared experimentally in a standard drilling operation. Thrust force and drill bit temperature were strongly correlated for each drill. Vibration values and noise level, which also had an influential relationship, were in the acceptable range for all experiments.

Many attempts have been made to investigate the relationship between thrust force and drill bit geometry^3^, drilling feed rate and rotational speed effects on thermal necrosis^10^, and breakthrough detection with thrust force measurements^12^. However, no research has compared different drills in terms of vibration, noise, temperature effects, thrust force, and speed deviation characteristics.

The highest thrust force and induced temperature of the drill bit were obtained with Drill 3 in the experiments. The relationship between induced temperature and thrust force was investigated in a research by Bachus *et al*^13^, and our empirical evidence after performing experiments also supports the past literature. Therefore, the importance of drill cooling during drilling in surgical operations is emphasised. Thermal necrosis depends not only on the temperature but also on the duration of heating. For example, a temperature value of 70°C could cause immediate damage, while a lower temperature value of 55°C for a 30 s duration could cause irreversible bone cell death^8, 14^. Induced temperature on the drill bit was measured between 78.11°C and 89.77°C. This shows that the heat increase in the drill bit during drilling could easily pass the levels for thermal necrosis. In addition, drill diameter, design, and bone density will affect the temperature^7, 15^.

In our study, significant differences were found between vibration measurements. The lowest vibration values were measured for Drill 3, and the highest value was found in Drill 2. Hand-transmitted vibration is associated with various vascular, neurological, and musculoskeletal disorders, collectively called HAVS. The course of HAVS is not clear, with different signs and symptoms recognised by different experts and in different countries. However, some disorders, especially vibration-induced white finger and neurological effects of hand-transmitted vibration, are widely recognised^5^. Although a significant difference was noted between vibration measurements, vibrations of the three drills were within the safe range. The daily exposure action value standardised to an 8-hour reference period should be 2.5m/s2.5. In our study, the highest measured value was 1.9376m/s2, which is lower than the standardised daily exposure value.

Noise level measurements indicated significant deviation in each drill. The best performance was obtained for Drill 1, while the highest noise was measured with Drill 2 as 39.807 dB. Exposure to high noise has negative effects on human health. The preliminary effect is hearing loss. In addition, hypertension, tachycardia, increased stress, and related effects can also occur^16^. A previous study reported that the risk of hearing loss may be present due to long-term high-volume exposure among orthopaedic surgery staff^6^. Surgical equipment, such as plaster drills, saw, oscillator, and reamer are among the sound sources in the orthopaedic surgery room. The recommended maximum noise exposure dose during an eight hours working day to prevent hearing loss is 90 decibel (dB)^6^. In our study, the noise level of the three drills was below this threshold.

Drills are electromechanical equipment and have nonlinear time variance dynamics according to temperature, unmeasurable processes, sensor noise, etc. Especially in cordless drills, drilling performance also depends on state of charge of the battery. When the charge in the battery decreases, the speed and torque produced by the drill decrease^17^. Speed deviation for Drill 1 and Drill 3 was almost negligible, but a significant deviation was observed for Drill 2. Drill 1 and Drill 2 have a built-in speed controller to overcome the effect of load changes on speed deviation. For an orthopaedic drill, speed control minimises drill speed change due to increased cutting load and frictional load of the drill bit in different tissues.

This study has some limitations. Experiments were performed in vitro. To standardise the experiments, identical artificial bones were used; therefore, histological changes during drilling could not be investigated. Further studies are warranted to confirm our experiments in vivo.

## Conclusion

Thrust force and drill bit temperature are correlated. A high thrust force may be an advantage as the surgeons are able to feel the bone transition by sensing the thrust force deviation. However, a high trust force may cause bone necrosis due to increased temperature. Noise level and vibration measurement are correlated. Although a high noise level could be a potential source of distraction, the noise levels of all drills are in the acceptable range. Moreover, all drills could be used in long operations with a negligible risk of HAVS. Speed deviation can be used to estimate the status of the drill bit when there was transition between bone sections, such as cancellous, spongy bone, or medulla. The two orthopaedic drills have almost similar characteristics, except thrust forces. Although the performance of the commercial drill is slightly behind its counterparts, it is still within acceptable range for all criteria. However, an open question that needs an answer is how to sterilise a commercial drill with known sterilisation procedures.
